# Case-control study of disease determinants for non-typhoidal *Salmonella *infections among Michigan children

**DOI:** 10.1186/1756-0500-3-105

**Published:** 2010-04-16

**Authors:** Muhammad Younus, Melinda J Wilkins, Herbert D Davies, Mohammad H Rahbar, Julie Funk, Chau Nguyen, Azfar-E A Siddiqi, Seongbeom Cho, Mahdi Saeed

**Affiliations:** 1 Department of Epidemiology, Michigan State University, East Lansing, Michigan 48824, USA; 2 National Food Safety and Toxicology, Michigan State University, East Lansing, Michigan 48824, USA; 3 Large Animal Clinical Sciences, Michigan State University, East Lansing, Michigan 48824, USA; 4 Communicable Disease Division, Michigan Department of Community Health, 201 Townsend Street, Lansing, Michigan 48913, USA; 5 Departments of Pediatrics and Human Development, Michigan State University, East Lansing, Michigan 48824, USA; 6 Division of Epidemiology, The University of Texas School of Public Health at Houston, Houston, Texas 77030, USA; 7 East 235 42ndStreet, (MS:150-3-78), Safety and Risk Management, Pfizer Inc, New York, NY 10017

## Abstract

**Background:**

Infections with *Salmonella *serotypes continue to be a significant global public health problem. In addition to contaminated foods, several other sources contribute to infections with *Salmonella *serotypes. We have assessed the role of socioeconomic factors, exposure to food, and environmental sources in the etiology of non-typhoidal *Salmonella *infections in Michigan children.

**Findings:**

A case-control study among Michigan children aged ≤ 10 years was conducted. A total of 123 cases of children with laboratory-confirmed *Salmonella *infections and 139 control children, who had not experienced symptoms of gastrointestinal illness during the month prior to the interviews, were enrolled. The cases and controls were matched on age-category (<1 year, 2-<6 years and 6-10 years). Data on socioeconomic status, food intake, and environmental exposures, were collected on the queried case and control subjects. After adjusting for race and household-income the final regression multivariable model revealed that *Salmonella *infections were significantly associated with attendance of a daycare center (adjusted matched odds ratio = 5.00, 95% CI: 1.51 - 16.58), contact with cats (MOR = 2.53, 95% CI: 1.14 - 5.88), and contact with reptiles (MOR = 7.90, 95% CI: 1.52 - 41.01), during the 3 days prior to the onset of child's illness.

**Conclusions:**

Study results suggest that exposure to environmental sources may play an important role in sporadic infections with *Salmonella *serotypes in children. Additional efforts are needed to educate parents and caretakers about the risk of *Salmonella *transmission to children from these sources.

## Findings

Epidemiologic investigations, including reports from the CDC's FoodNet, have demonstrated higher incidences of *Salmonella *infections in children compared to adults [1,2]. In 2003, the incidences of laboratory-confirmed cases of *Salmonella *infections reported at FoodNet sites were 122.7 cases per 100,000 population for children aged < 1 year, 50.6 cases per 100,000 population for children aged 1-4 years, and 10.8 cases per 100,000 population for those aged ≥ 5 years [3]. During 2002-2006, Michigan Department of Community Health (MDCH), reported 63.96 cases of salmonellosis per 100,000 population for children aged < 1 year, 12.62 cases per 100,000 population for children aged 1-9 years, and 8.45 cases per 100,000 population for aged ≥ 10 years [4]. One of the reasons of higher incidence of salmonellosis cited in the literature is the increased case detection of *Salmonella *infections in children compared to the adult population. In young children with gastrointestinal symptoms, increased detection is likely due to: 1) the parents will seek medical attention and 2) the healthcare provider will more likely submit a sample for culture [5].

The majority of sporadic cases of salmonellosis in the adult population results from consumption of contaminated food and traveling to areas where *Salmonella *is more prevalent [6]. However, risk factors for salmonellosis in children have not been extensively evaluated in population-based epidemiologic studies. The majority of the studies in children have been conducted in response to salmonellosis outbreaks, particularly in daycare centers and nurseries. The risk factors identified in outbreaks may not be similar to those of sporadic cases of salmonellosis since exposures in sporadic cases vary widely. A few studies have identified eating undercooked eggs [7], infant formula [1], attending a day care with a child with diarrhea [1], household member with a gastrointestinal (GI) symptoms [7], and contaminated home environment [8] as risk factors for *Salmonella *infections in children. It has been suggested that contaminated environmental sources contribute more than contaminated food vehicles in the acquisition of *Salmonella *infections in children [7]. This report describes a population-based case-control study conducted to identify potential risk factors for sporadic, non-typhoidal *Salmonella *infections in Michigan children aged ≤ 10 years.

## Methods

### Study design and subjects

A case-control study was conducted between December 15, 2006 and October 15, 2007 among Michigan children aged ≤ 10 years. Salmonellosis is a notifiable disease under the Michigan Communicable Disease Rules [9]. Therefore, physicians and laboratories across Michigan are required to report cases of salmonellosis to their local health department (LHD) in either the jurisdiction where the individual with suspected salmonellosis resides or where the reporting facility is located (passive surveillance system). In addition to reporting to the MDCH, LHDs and clinical laboratories send isolates to the MDCH Bureau of Laboratories for serotyping and the results are entered into the state wide Michigan Disease Surveillance Systems (MDSS).

In this study, cases were defined as children aged ≤ 10 years with laboratory-confirmed *Salmonella *infections with permanent Michigan address. Case children were excluded from the study if the case 1) was reported as part of a salmonellosis outbreak investigated by Michigan Department of Community Health (MDCH) officials and 2) had a reported congenital malformation (e.g., birth defect), serious medical condition, or a concomitant infection.

Controls were children aged ≤ 10 years who were not diagnosed with any enteric infections (e.g., salmonellosis, campylobacteriosis) by a healthcare provider and did not experience any enteric disease symptoms (e.g., diarrhea, vomiting, nausea) during the 30 days prior to the interview day. Controls were excluded if the child had a congenital malformation or serious medical condition. Controls were enrolled by two methods: 1) case parent(s) were asked to identify a child of similar age to their own child and 2) using the on-line telephone directory http://www.whitepages.com. This directory has a reverse address function that allowed the compilation of lists of potential control household phone numbers. Households were called, and after explaining the study's objective, we asked if there were any children aged ≤ 10 years living in the household. Consented parents or caretakers of these children were interviewed.

### Questionnaire and data collection

Data was collected using a structured questionnaire on sociodemographic characteristics (e.g., age and sex, household income, parental education), child feeding practices (e.g., breast feeding, formula milk, use of pacifier), child rearing (e.g., daycare, pre-school, or elementary school attendance), food exposures (e.g., consumption of milk, meat, poultry, eggs, produce, water) and various environmental exposures (e.g., contact with animals, contact with a person having GI symptoms). The questionnaire is presented under additional file 1.

Exposures were assessed during the 3 days preceding the illness onset date for cases, and the 3 days prior to the interview day for controls. The 3 days exposure experience encompasses the common incubation period for salmonellosis and has been used in other similar studies [10,11]. Parents or caretakers were given the option to fill out a self-administered mail-in questionnaire or participate in a 15 to 20 minute phone interview with a trained interviewer. The questionnaire was pilot-tested on volunteer parents, issues identified in this exercise were addressed, and appropriate changes were incorporated into the final version of the questionnaire. An informed consent was obtained from parents or caretaker prior to administering the questionnaire. The study protocol was reviewed and approved by the IRBs at Michigan State University and MDCH.

### Data management and statistical analysis

An age-matched (<1 year, 2-<6 years, and 6-10 years) pair univariable analysis of cases and controls using the conditional logistic regression was performed to examine associations between *Salmonella *infections and hypothesized risk factors. A multivariable analysis was conducted to identify factors associated with *Salmonella *infections, simultaneously controlling for potential confounders including race and household income, and matched odds ratio (MOR) and respective 95% confidence interval (95% CI) was computed for each predictor variable. All variables with a p values < 0.25 on univariable analyses, along with those hypothesized risk factors for *Salmonella *infections, were initially considered for inclusion in the multivariable model. After identifying the correlated variables, we examined the impact of collinearity by separately entering the variables (e.g., income, education, and race) into the multivariable regression model. We used the backward elimination method to obtain a parsimonious but yet plausible model. Additionally, to investigate potential effect modification, we also included several two way interaction terms in the model, 'household income and race,' 'household income and reptile ownership,' and 'and reptile ownership. A p-value < 0.05 was considered statistically significant in all analysis. SAS was used to analyze the data.

Population attributable risk (PAR) for selected variables were estimated from the final multivariable model using Levin's formula for the calculation [12].

## Results

A total of 123 cases representing a participation rate of 72.78% (123/169) were enrolled. A total of 139 control children were enrolled using one of the following two methods. Additional files 2 and 3 describe the enrollment of cases and controls respectively and list the number and type of exclusion criteria that were encountered during this study.

The majority (85%) of the cases were interviewed within 20 days from the date when their records were available in the MDSS database. Control child was enrolled in parallel with cases enrollment however the cases were not individually matched with controls on the time of enrollment.

Comparison of the enrolled cases and controls is presented in Table 1. The enrolled cases and controls did not differ by socioeconomic and demographic characteristics including parental education, and annual household income except for distribution of racial composition (*p *< 0.01).

**Table 1:** Socioeconomic characteristics of children aged ≤ 10 years enrolled in a population-based case-control study to identify risk factors for *Salmonella *infections, Michigan, 2007.

Socioeconomic characteristics	Cases n = 123		Controls n = 139		*p*-value*
	No.	(%)	No.	(%)	
**Age (year)**					0.54
< 1	22	(17.89)	25	(17.99)	
1-<6	66	(53.66)	66	(47.48)	
6-10	35	(28.46)	48	(34.53)	
					
**Sex**					0.86
Female	66	(53.66)	76	(54.68)	
Male	57	(46.34)	63	(45.32)	
					
**Race**					< 0.01*
Caucasians	107	(87.70)	92	(67.15)	
African-Americans	9	(7.38)	27	(19.71)	
Other minorities (e.g., Asian, Alaskan Indian)	6	(4.92)	18	(13.14)	
					
**Parental education**					0.94
Elementary to High school	30	(24.39)	33	(23.91)	
Some college to college degree	72	(58.54)	85	(61.59)	
Post-graduate degree	19	(15.45)	18	(13.04)	
Refused to answer	2	(1.63)	2	(1.45)	
					
**Annual income household**					0.34
<$ 35,000	26	(21.31)	25	(18.12)	
$35,001- $50,000	17	(13.93)	17	(12.32)	
$50,001- $75,000	28	(22.95)	39	(28.26)	
>$75,000	38	(31.15)	33	(23.91)	
Refused to answer	13	(10.66)	24	(17.39)	
					
**Area of residence**					0.31
High income: $>60000	31	(25.20)	35	(25.18)	
Medium income: $38000 - $60000	56	(45.53)	74	(53.24)	
Low income: $<38000^δ^	36	(29.27)	30	(21.58)	

*significant at *P *< 0.05. P-value were computed using chi-square test

^δ ^Categorized based on zip code level median household income obtained from the US Bureau of Census, 2000.

Tables 2 and 3 show the results of univariable and multivariable logistic regression models respectively. The final multivariable model after adjusting for race, household income, and other known risk factors for salmonellosis [contact with a person having GI symptoms and other food related exposure (eating egg/meat/poultry) in the past 3 days] revealed that *Salmonella *infections were significantly associated with attending a daycare center (MOR = 5.00 (95% CI: 1.51-16.58), contact with cats (MOR = 2.53 (95% CI: 1.14-5.88), and contact with reptiles (MOR = 7.90 (95% CI: 1.52-41.01). None of the two-way interactions tested (household income and race, household income and reptile ownership, and race and reptile ownership) were statistically significant (p > 0.05). Reptile exposure had the highest PAR% (35.61) among the risk factors identified for salmonellosis in our study.

**Table 2:** Univariable analyses of putative risk factors for *Salmonella *infections in children aged ≤ 10 years, assessed in a population-based case- control study, Michigan. 2007.

Variables	Cases n = 123		Controls n = 139		Unadjusted MOR* (95% CI**)
	No.	(%)	No.	(%)	
**Household related variables**					

**Number of people in household**					
≤ 4	82	(66.67)	77	(55.40)	Reference
> 4	41	(33.33)	62	(44.60)	1.56 (0.94-2.59)
**Number of children aged ≤10 years**					
1	40	(35.52)	58	(41.73)	Reference
2-3	73	(59.35)	72	(51.80)	1.47 (0.87-2.46)
≥ 3	10	(8.13)	9	(6.47)	1.53 (0.57-4.01)
**Number of bedrooms**					
> 3	36	(29.27)	67	(48.20)	Reference
2-3	70	(56.91)	46	(33.09)	2.75 (1.59-4.78)
≤ 2	17	(13.82)	26	(18.71)	1.19 (0.57-2.47)
**Family room flooring**					
Wood	17	(13.82)	12	(8.63)	Reference
Carpet	88	(71.54)	117	(84.17)	0.52 (0.24-1.14)
Other and combination	18	(14.63)	10	(7.19)	1.26 (0.43-3.61)
**Attend a daycare**					
No	106	(86.18)	130	(93.53)	Reference
Yes	17	(13.82)	9	(6.47)	2.28 (0.98-5.32)
**Child attends school other than daycare**					
No	69	(56.10)	72	(51.81)	Reference
Yes	54	(43.92)	67	(48.20)	0.96 (0.49-1.90)

**Food related exposures**					

**Ate eggs/egg containing product**					
No	44	(40.74)	60	(44.44)	Reference
Yes	64	(59.26)	75	(55.56)	1.14 (0.65-1.98)
**Ate poultry**					
No	13	(11.30)	17	(12.50)	Reference
Yes	78	(67.83)	95	(69.85)	1.07 (0.49-2.34)
**Ate poultry at:**					
Home	15	(41.67)	78	(56.52)	Reference
Outside home at a restaurant	3	(8.33)	3	(2.17)	0.94 (0.35-2.55)
Both home and outside home	4	(11.11)	10	(7.25)	3.37 (0.86-13.21)
**Ate meat**					
No	36	(30.51)	51	(37.78)	Reference
Yes	58	(49.15)	60	(44.44)	1.31 (0.78-2.40)
**Ate meat at:**					
Home	9	(25.00)	46	(33.33)	Reference
Outside home at a restaurant	2	(5.56)	6	(4.35)	1.05 (0.31-3.53)
Both home and outside home	4	(11.11)	4	(2.90)	1.36 (0.32-5.20)

**Univariable analysis**	**Cases**		**Controls**		Un-adjusted MOR* (95% CI**)
	**n = 123**	**(%)**	**n = 139**	**(%)**	

**Drinking water source**					
Bottled	25	(20.33)	38	27.34	Reference
Municipal tap	73	(59.35)	81	(58.27)	1.01 (0.62-1.71)
Private well water	25	(20.33)	20	(14.39)	1.542 (0.70-2.89)

**Family Kitchen practices**					

**Keeps eggs refrigerated**					
Always	118	(95.93)	136	(97.84)	Reference
Never or sometimes	5	(4.07)	3	(2.16)	1.92 (0.45-8.22)
**Clean kitchen counters with**					
Soap and disinfectant	45	(36.89)	70	(50.36)	Reference
Soap and water only	26	(21.31)	25	(17.99)	1.56 (0.84-3.03)
Disinfectant only	51	(41.80)	44	(31.65)	1.75 (1.01-3.04)
**How often clean kitchen counter**					
Daily	109	(90.08)	130	(93.53)	Reference
More than once a week/once a					
Week/less than once a week	12	(9.92)	9	(6.47)	1.51 (0.61-3.73)

**Other environmental exposure**					

**Handled packages of raw meat/eggs while shopping with child**					
Did not go to shopping with child	64	(52.03)	87	(62.59)	Reference
Handled packages with plastic/gloves	15	(12.20)	16	(11.51)	1.26 (0.54-2.76)
Handled packages without plastic/gloves	44	(35.77)	36	(25.90)	1.62 (0.94-2.90)
**Contact with a person having GI upset**					
No	96	(78.05)	123	(88.49)	Reference
Yes	27	(21.95)	16	(11.51)	2.15 (1.10-4.21)
**Contact with animal**					
No contact with any animal	42	(34.15)	81	(58.27)	Reference
Any animal contact	81	(65.85)	58	(41.73)	2.63 (1.59-4.34)
Dogs	53	(43.09)	47	(33.81)	1.45 (0.88-2.39)
Cats	35	(28.46)	21	(15.11)	2.23 (1.22-4.11)
Reptiles	17	(13.82)	5	(3.60)	4.16 (1.49-11.62)
Birds	4	(3.25)	1	(0.72)	4.57 (0.50-41.33)
Hamster	1	(0.81)	2	(1.44)	0.68 (0.06-7.70)

**Travel history**					

No	90	(73.17)	106	(76.26)	Reference
Yes	31	(25.20)	33	(23.74)	1.08 (0.61-1.91)

*Matched Odds ratio calculated using conditional logistic regression, ** Confidence interval. All exposure data were gathered for during the 3 days of child's illness onset for cases and 3 days before the interview for controls.

**Table 3:** Multivariable analysis of putative risk factors for *Salmonella *infections in children aged ≤ 10 years, assessed in a population-based case-control study, Michigan, 2007.

Variable	Unadjusted MOR^a^ (95% CI^b^)	Adjusted MOR (95% CI^b^)	PAR^C^
Contact with cats			
No	Reference	Reference	
Yes	2.23 (1.22-4.11)	2.53 (1.14-5.88)	24.29 (4.32-50.73)

Contact with reptiles			
No	Reference	Reference	
Yes	4.16 (1.49-11.62)	7.90 (1.52-41.01)	35.61 (4.0-76.20)

Attended a daycare			
No	Reference	Reference	
Yes	2. 28 (0.98-5.32)	5.00 (1.51-16.58)	28.36 (4.80-60.72)

^a ^Matched odds ratio (calculated using conditional logistic regression analysis), ^b ^Confidence interval, ^C ^Population attributable risk

Age-group (<1 year, 2-<6 years and 6-10 years) matched analysis between cases and control was performed. Model was adjusted for race, household income, and other known risk factors for salmonellosis [contact with a person having GI symptoms and other food related exposure (eating egg/meat/poultry)]. Exposure data were gathered for the 3 days prior to child's illness onset for cases and 3 days before the interview for controls

## Discussion

We found that cases of laboratory-confirmed salmonellosis were associated with several potentially modifiable risk factors and thus findings of this study can be used for enhancing salmonellosis prevention efforts in Michigan. Concurring with other investigations, our data suggest that children attendance of a daycare significantly increases the odds of *Salmonella *infections. Infections with several enteric agents have been associated with attending daycare [8,12,13]. The spread of infections in daycare centers is facilitated by crowding and microbial contamination of the environment [14]. Simple measures to control and prevent infections such as washing hands with soap and water after changing diapers, after assisting children with the toilet, and before handling food would help to substantially reduce the incidence of infections related to daycare.

We have found that contact with animals including reptile was associated with significant risk for salmonellosis in Michigan children. Salmonellosis is a well-recognized bacterial zoonoses [15]. Animals are the predominant reservoirs for the bacteria, and the prevalence of *Salmonella *carriage varies by species [16]. An estimated 90% of all reptiles including turtles and iguanas, carry and shed *Salmonella *in their feces intermittently [17]. *Salmonella *serotypes have been isolated from most vertebrates including dogs and cats with carriage rates of up to 36% and 18%, respectively [18]. However, a much higher (up to 94%) *Salmonella *carriage rate has been observed in reptiles and amphibians [19]. In a recent case-control study conducted using FoodNet sites, reptile ownership was associated with more than 5-fold increased risk of salmonellosis in children aged < 1 year [1].

In contrast to other similar studies [20] that reported an association between reptile-associated *Salmonella *specific serotypes (e.g., *S*. Stanley, *S*. Poona, *S*. Litchfield) and contact with reptile, our study did not find such association. This could be because of the limited sample size when the data were stratified by serotypes. We did not find significant differences in the frequency of *Salmonella *serotypes isolated from case children exposed to only reptiles or only cats. A recent study on *Salmonella *serotypes in captive reptiles reported a high diversity of *Salmonella *serotypes among reptile, which did not include serotypes Stanley, Poona or Litchfield [21]. Additionally, the MDCH registry-based study classified 59 isolates of *Salmonella *serotypes associated with children cases to be reptile-associated. The classical reptiles-associated *Salmonella s*erotypes were not reported [22]. In this study, we found serotype Enteritidis and Typhimurium, which are frequent contaminants of chicken and meats, to be common among case children with reported contact with reptiles or cats. These findings are consistent with the previous report [22]. This assumed cycle of transmission may have resulted from foods contaminated with these serotypes that was fed to these animals [21].

Food-related exposures such as consumption of chicken, meat, and eggs/egg-containing product (established risk factors for salmonellosis in adults) did not show a significant association with *Salmonella *infections. It is conceivable the actual magnitude of food exposure related risk for *Salmonella *infections was not demonstrated in this study due to several factors. First, we obtained exposure information from interviewing surrogate sources (i.e., parents or caretakers). Secondly, certain exposures such as contact with animals are more likely to be accurately recalled, but the consumption of specific foods is more difficult to recall and thus prone to measurement error.

Exposure to contaminated environment was more commonly reported than exposure to contaminated food vehicles as a possible source for *Salmonella *infections in children [7,23]. Results of this study are in agreement with the findings of a case-control study conducted in the UK, where none of the household-related variables showed significant associations with *Salmonella *infection [24]. A recent study reported that children aged < 1 year who ride in a shopping cart with meat or poultry placed next to them have a 4-fold increased risk for salmonellosis [1]. Although some studies [8,24] have reported the isolation of *Salmonella *from household dust, soil samples near the home, and samples from bathrooms, we have not microbiologically evaluated contamination of the household environment.

This was the first population-based case-control study in Michigan conducted to identify risk factors for salmonellosis in children. Although not all reported cases were enrolled in the study, participants and non-participants were drawn from the same base population, and the two groups were similar with respect to demographic characteristics (p > 0.05). Our data were limited to laboratory-diagnosed cases only, and thus are biased (reporting bias) by factors that affect the probability of an illness being reported; caretakers of case children with mild to moderate disease symptoms may not seek medical care (selection bias) [5]. Further, recall bias may be present in the measurement of some exposure variables. Parents knew the disease status of their children prior to the interview and this may have influenced their responses [25]. We did not obtain specimens for culture from controls to exclude asymptomatic cases of salmonellosis. It is possible that some of our control children were asymptomatic *Salmonella *carriers and thus would have been misclassified. However, given the very low (1%) prevalence of chronic carriers of *Salmonella *in healthy populations, we expect minimal misclassification due to this phenomenon [26].

## Conclusions

This study revealed that attending a daycare center and contact with cats and reptiles within three days before a child's illness onset are important risk factors for infection with *Salmonella *serotypes in Michigan children. Data suggest that the contribution of environmental sources plays an important role in the acquisition of *Salmonella *infections in children. Further efforts are needed to educate parents and caretakers about the risk of *Salmonella *transmission to children from cats and reptiles, in addition to individuals having GI symptoms.

## Competing interests

The authors declare that they have no competing interests.

## Authors' contributions

MY and AMS designed the study, conducted data analyses, and led the writing of the manuscript. MJW and HDD contributed to the study design and final data analyses. MHR, AAS and JF contributed to the data analysis and evaluation of results. CN participated in the data evaluation and coordination. SC and AMS oversaw the project, and helped in writing and the completion of the manuscript. All authors reviewed and approved the manuscript.

## Supplementary Material

Additional file 1 **Study questionnaire**. This questionnaire contains demographic, exposure including food intake information before the onset of the disease for cases and before the interview for control. Click here for file

Additional file 2 **Michigan *Salmonella *case-control study, 2007: Enrollment of cases (12/15/06 - 10/15/2007)**. All cases of children 10 years or younger in Michigan who were confirmed by laboratory methods to be a case of non-typhoidal *Salmonella *infection and signed a consent form, were enrolled. Click here for file

Additional file 3 **Michigan Salmonella case-control study, 2007: Enrollment of controls (12/15/06 - 10/15/2007)**. All non-case children who were matched on age and gender with cases and signed a consent form were enrolled. Click here for file
