# Supplementary material for: Case-control study of disease determinants for non-typhoidal Salmonella infections among Michigan children
Source: BMC Res Notes. 2010 Apr 16;3:105. doi: 10.1186/1756-0500-3-105 (PMC2862038; doi:10.1186/1756-0500-3-105)
Supplement: Additional file 1 — Study questionnaire. This questionnaire contains demographic, exposure including food intake information before the onset of the disease for cases and before the interview for control. [file 1756-0500-3-105-S1.DOC]

# Interview starts (time)----------- Interview ends (time)---------------

# Michigan *Salmonella* Case-Control Study

**STUDY QUESTIONNAIRE**

[Telephone interview Form]

**Study ID:__________________________________**

**Interviewer:___________________________**

**Date of interview:________\_______\__________**

**Study Introduction:**

Hello, my name is _________________________________ and I work for Michigan State University (MSU).

Are you the parent or guardian of *(___________)*?

*Insert child’s name*

MSU is conducting a study, in collaboration with the Michigan Department of Community Health (MDCH), to identify factors and conditions that make some children more likely than others to get *salmonellosis*, a foodborne illness. Children are also at a higher risk of getting salmonellosis compared to adults. Therefore, we are trying to study the causes of this higher infection rate. *Salmonella* infection is a reportable disease by law in Michigan. Your contact information was obtained with the permission of our collaborator, Michigan Department of Community Health.

We are very hopeful that you will be willing to participate in this project to enable us to generate very much needed information on the conditions associated with the disease in Michigan children.

Your participation is voluntary. However, we are asking for your help because the knowledge gained through this study may contribute to the control and prevention efforts of *Salmonella* infections in Michigan’s children.

The type of effort needed from you, as a participant, is to complete a short questionnaire. You can answer the questions over the phone or by filling out the questionnaire mailed to you. There are no known physical and/or psychological side effects associated with these questions. The questionnaire will only take about 10 minutes for older children, and 15-20 minutes for younger ones.

All information gathered from you will remain confidential. Data will be reported in a summary form and no individually identifiable responses will be presented or published. You may decide to withdraw from the study even after the interview, and you can decline to answer any question that makes you uncomfortable. Do you have any questions?

**Are you willing to take part in this research?** _____Yes “Thank you in advance for your contribution to this project. “

_____No “Thank you for your time”

**Singed consent: Yes No**

**Verbal consent Yes No**

(Please read the consent form over telephone)

**How would you like to fill out the questionnaire ____ phone or by ________mail?**

_____By mail (Confirm the address):___________________________________

_____**By phone interview**

**Is now a good time to talk to you? _____**Yes “Thanks, we will now begin the questionnaire”

_____No “When can I call you back?

Day and date: _____________ Time: ______

**Eligibility Criteria:** **To determine the eligibility of (_____________) for this study, could you please tell us if (_____________) has any serious medical conditions (e.g cancer: leukemia, lymphoma) or birth defects?**

______**Yes** [We apologize, we cannot enroll (___________) as a participant in this

particular study because having a serious medical condition will complicate the understanding of *Salmonella* infection risk factors.

______**No**  [please proceed with the interview]

##### DEMOGRAPHIC INFORMATION

### Below is the information that will be obtained from the MDSS* database

### (If NOT available from case report form, please ask parent/guardian)

NOTE: fill in dates prior to illness onset below, before starting interview

**1) Illness Onset date ______/______/______ (dates three days prior to illness onset ___/____ to ____/____)**

*For example illness onset 7/17/2006 so three days prior would be 7/14 – 7/16, use the above dates throughout questionnaire*

*For controls for this case, use the three-day period prior to interview date throughout the questionnaire*

**2) Child’s name _______________________________**

**3) Age of the child at time of illness onset** (in completed months): [[1]](#footnote-2) Days/Months/Years *(number of days if the child is less than a month)*

**4) Child’s gender: _____ Male ____ Female**

**5) Zip code of child’s permanent residence _______**

**6)** **County of child’s permanent residence** _______

**7) Child’s race**

**_____Caucasian\White**

**_____African American\Black**

**_____Asian**

**_____Pacific Islander**

**_____Unknown**

**8) Child’s ethnicity**

**_____Hispanic/Latino**

**_____Non-Hispanic/Latino**

**_____Unknown**

**_____Other (specify): __________________**

**9) Case is reported as part of an outbreak**

*(Only for cases)*

**_____Yes _____No _____Unknown**

Information for this part of a questionnaire will be obtained from a parent/guardian (preferably the mother) of the child. Respondent must be a parent or guardian, or permission must be granted from a parent or guardian for someone else (nanny, grandparent, step parent) to provide this information.

**10) What is your relationship to** ***(_______________)*:**

*child’s name*

**_____Mother**

**_____Father**

**_____Other (with parent or guardian’s permission)**

**HOUSEHOLD INFORMATION**

### First, I will ask some questions about your household during the 3 days prior to (_______) illness, from (______to_______)

Child’s name insert dates from Q 1

**[For cases: All questions should refer to the 3 days preceding the illness onset date]**

**[For controls: All questions should refer to the 3 days time preceding the interview date]**

**11) How many people live in your home? _______**

**12) How many children in your home are less than 10 years of age? _______**

13) How many children in your home are in diapers? _______

**14) How many bedrooms are in your house? ________**

**15) What kind of flooring do you have in the family room?**

**_____Carpet**

**_____ Wood**

**_____Tile/linoleum**

**_____Does not have a family room**

**_____Other (specify):________________________________ indicate rugs here**

**16) What kind of flooring is in (______________) bed room?**

*child’s name*

**_____Carpet**

**_____ Wood**

**_____Tile/linoleum**

**_____Other (specify):______________________________indicate rugs here**

##### CHILD CARE

Now, I will ask you about (___________) childcare during the 3 days prior to

(his/her) illness, from (______to_______). (insert same date’s as above)

**17) Does** (____________) **attend a day care outside of your home?** ____Yes _____No *(If No, go to Q#18)*

**17a) How many hours per week does (*he/she*) usually spend in day care?**

_______ hours/wk

###### 17b) How many total children attend (_____________) daycare? _____ children

*Child’s name*

###### 17c) About how many children share the same room as (_____________)?

###### _____ children *Child’s name*

**17d) About how many children in your child’s room are in diapers? _______**children

**17e) About how many day care workers attend to this room? _____** workers

**17f) Is there a separate room for changing diapers in the day care?**

**17g) Is there a sink with soap and water next to the diaper-changing area in the**

**day care?**

__Yes __No _Don’t know

**17h) In the day care, approximately how far in feet is the diaper-changing area from**

**the area where food, milk, and other beverages are handled?** ______ft

**17i) Are you aware of any child at the daycare who experienced vomiting, diarrhea,**

**or abdominal cramps during the 3 days prior to *(__________)* illness?**

____Yes _____No _____Don’t know *(If No or Don’t know, go to Q #17k)*

**17j) How many children had nausea, vomiting, diarrhea or abdominal cramps**

**during the 3 days prior to *(__________)* illness? ______**children

*Child’s name*

**17k) Who usually prepares the food *(child’s name)* eats while at the daycare?**

*(Mark all that apply)*

**_____Mother**

**_____Father**

**_____Other family member**

**_____Daycare personnel**

**_____Other (specify):_____________________________________**

**18) Does (*___________*) attend a preschool, kindergarten, or elementary school?** _____Yes _____No  *(If No, go to Q #19)*

*Can be in addition to daycare – such as before or after school care programs.*

**18a) Who prepares the food that (____________) eats while at school?**

***(Check all that apply)***

**_____Mother**

**_____Father**

**_____Other family member**

**_____Cafeteria/cook**

**_____Other (specify): _________________**

###### 19) Did you take *(______________)* with you while grocery shopping during the 3 days prior to (his/her) illness?

_____Yes _____No ____Don’t know *(If No or don’t know, go to Q #20)*

## 19a) Did you use gloves or plastic bags when handling packages of raw chicken, meat, and egg products while grocery shopping that time?

____Yes ____No ____ Don’t know __ Did not handle meat or egg products

##### FOOD HISTORY OF THE CHILD

### This part of the questionnaire asks you about (child’s name) food history and activities (skip if over age 1 year)

## 20) Did you put (*______________*) on the floor or carpet without a blanket during the 3 days prior to *(___________)* illness?

______Yes _____No *(If No, go to Q #21)*

## 20a) About how often was *(____________)* placed on (or played on) the floor or carpet without a blanket in the 3 days prior to his/her illness?

**_____Never**

**_____Once a day**

**_____More than once a day**

_____**Other (specify):___________________________________**

21) Was (­­­­­_________) breast-fed during the 3 days prior to (his/her) illness? _____Yes _____No _______Don’t know

22.) Did you use formula to feed (_________) during the 3 days prior to (his/her) illness?

____Yes _____No ___Don’t know (If No or Don’t know, go to Q# 23)

**22a) What type (e.g., milk, soy, rice-based) and brand of formula did you feed (*child’s name*) during the 3 days prior to (*his/her)* illness?**

**____________________________________________________________________** Please record exact brand and type if known.

If not known, use list below to prompt recall.

*(Check all that apply)*

## _____Isomil

## ____ Enfamil

## _____Bright Beginnings

## _____Nestle

## _____Similac

## _____Store brand (e.g. Meijers, Krogers etc)

____ **Other (specify):** ____________________

## 23) Did *(_____________)* use a pacifier during the 3 days prior to (*his/her*) illness?

## ____Yes _____No ___Don’t know

**24)** **Did *(____________) eat egg during the 3* days prior to (*his/her)* illness?**

­­­­____Yes (*If yes, how it was prepared? ____ fully cooked* ____ *partially cooked*)

_____ No _____Don’t know

**25) Did (***_______________***) eat any food that contained eggs during the 3 days prior to (*his/her)* illness?**

## ____Yes _____No ___Don’t know

**Food History**

*(Skip if less than 1 year of age and go to Q#27)*

**26) Did (____________) eat or drink any unpasteurized milk, or cheeses such as queso fresco made with unpasteurized milk during the three days before your illness?**

____Yes _____ Probably yes ____ Probably not _____No _____Don’t know

**26a)** **Did *(____________) eat egg during the 3* days prior to (*his/her)* illness?**

## *child’s name*

_____Yes *(If yes, how it was prepared? ____fully cooked­­­­­ ____half cooked ?)*

___No ___ Don’t know

## 26b) Did *(____________)* eat any food that contained eggs (such as: cookie dough, salad dressings, mayonnaise, ice cream, custard, cake mix) during the 3 days prior to (*his/her)* illness?

Yes ______ if yes, prepared at home: Yes _____ No ______

No_______ Don’t know ______

**26c)** **Did** *(____________)* **eat any food that contained poultry (such as chicken, or turkey) during the 3 days prior to (*his/her)* illness?**

Yes ______ if yes, prepared at home: Yes ______ No _____

## No_____ Don’t know ______

**26d)** **Did** *(____________)* **eat any food that contained meat other than poultry (such as hamburger) during the 3 days prior to (*his/her)* illness?**

Yes _____ if yes, prepared at home: Yes ____ No ____

## No­_______ Don’t know ______

**26e) In the three days before (__________) illness, did he/she eat at any of the following types of commercial food establishment?** *(mark all that apply)*

____Restaurant

If don’t remember then ask Q26 f and g

____Fast-food establishment

____Cafeteria

____Deli

____Read-to-eat food served in a supermarket or department store

____Street-vended food

____Concession stand at sporting event

____Snack bar

____Gas station

____Other (specify)

**26f) How often does (__________) eat at fast food restaurants?**

*child’s name*

_____Daily

_______More than once a week

_______Once a week

_______Once a month

_______Never

*Other(Specify):________________________________________*

**26g) What is (__________) preferred food at fast food places?**

*child’s name*

________Hamburger

________Chicken

________Other (specify

Question about source(s) of drinking water

## 27) Now I am going to ask about the types of water sources (*child’s name*) drank during the 3 days prior to (*his/her)* illness? Did *(child’s name)* drink water from:

Municipal tap water _____Yes _____No _____Don’t know

Private well water _____Yes _____No _____Don’t know

Untreated surface water _____Yes _____No _____Don’t know

(river, pond, lake)

## Bottled water _____Yes _____No _____Don’t know

Other:_________________________________________________

**INTRAFAMILIAL TRANSMISSION OF *SALMONELLA***

### This part of the questionnaire asks you about your family’s possible exposure to *Salmonella* during the 3 days prior to illness

**28) Was anyone in your household ill with symptoms of stomach upset, which may include nausea, vomiting, diarrhea, and abdominal cramps during the 3 days prior to (________) illness?**

____Yes _____No ____Don’t know *(If No or Don’t know, go to Q# 29)*

28a) Did (he or she) seek medical care for these symptoms?

____Yes _____No ____Don’t know (If No or Don’t know, go to Q29)

## 28b) What was the diagnosis? _____________diagnosis or ____Don’t know

## 29) During the 3 days prior to (___________*)* illness, did (*he/she)* visit any friends

## or relatives who had symptoms of stomach upset, which may include nausea, vomiting, diarrhea, and abdominal cramps?

## ____Yes _____No ____Don’t know

## 30) During the 3 days prior to (*______________)* illness, did anyone who had symptoms of stomach-upset visit your home?

##

## ____Yes _____No ____Don’t know

**FAMILY KITCHEN PRACTICES**

### This part of the questionnaire asks you about your family’s kitchen practices

31) Do you keep your eggs in a refrigerator?

_______Never _______Sometimes _____Always

**32) Do you wash your kitchen counters, sinks, and cutting boards after preparing raw chicken?**

_______Never _______Sometimes _____Always (If never go to Q#35)

**33) How do you clean your kitchen counters?**

­________with soap and water ________with a disinfectant

**34) How often do you clean your kitchen counters?**

_____Less than once a week

_____Once a week

_____More than once a week

_____Daily

# ANIMAL EXPOSURE

### This section of the questionnaire asks you about pets

35) During the 3 days prior to (___________) illness, did (he/she) have contact with any type of pet, your pet or someone else’s pet or animals in a petting zoo setting?

____Yes _____No ____Don’t know (If No or Don’t know, go to Q# 36)

35a) What kind of pet(s) did (___________) have contact with during the 3 days prior to (his/her) illness?

(Check all that apply, get as much detail as possible)

_____Dogs *(if yes, how many?) ____# Dog(s) age(s) ____weeks, months, adult*

_____Cat *(if yes, how many?) ____# Cat(s) age(s)*  *____weeks, months, adult*

_____Reptiles *(if yes, how many) ____# describe _____ (iguana, cornsnake etc)* _____Birds *(if yes, how many) ___# describe _____________________ (chicken, duckling, parakeet etc.)*

_____Hamster

_____Gerbil

_____Ferret

_____Other (specify): ______________________________________________________________

# 35b) Were any of these animals noticeably ill with diarrhea?

# ____Yes _____No ____Don’t know

##### TRAVEL HISTORY

### This section of the questionnaire asks you about your child’s travel history

**36) Did (________________) travel anywhere during the 3 days prior to (his/her) illness?**

____Yes _____No ____Don’t Know (If No or Don’t know, go to Q #37)

## 36a) Did *(_________________)* meet any person with symptoms of stomach upset during your visit?

##

## ____Yes _____No ____Don’t know

##

# SOCIOECONOMIC HISTORY

### Just a couple more questions about your income and education, you don’t need to answer if you are uncomfortable

**37) What is the highest level of education you have completed?**

_____Some High School

_____High School or GED

_____Some college or technical training

_____4 year college degree

_____Graduate degree

_____Post graduate degree

**37a) What is your total annual household income?**

_____ less than $20,000

_____$20,000 - $35,000

_____$35,001 - $50,000

_____$50,001 - $75,000

_____$75,001 - $100,000

_____more than $100,000

_____Refused to answer

“That’s it! Thank you so much for your time, we really appreciate that you have shared this important information with us as we try to research this important childhood disease

If you have any questions related to the study you may contact Dr. Mahdi Saeed, the principal investigator of this research, at 517-432-9517.”

-------------------------------------------------------------------------------------------------------------------------------

**Investigators:**

## Dr. Mahdi Saeed

Professor, Department of Epidemiology, College of Human Medicine

Michigan State University

Tel: 517-432-9517

E-mail: [saeeda@msu.edu](mailto:saeeda@msu.edu)

1. *Michigan Disease Surveillance System maintained by the Michigan Department of Community Health [↑](#footnote-ref-2)
