# Supplementary material for: Case-control study of disease determinants for non-typhoidal Salmonella infections among Michigan children
Source: BMC Res Notes. 2010 Apr 16;3:105. doi: 10.1186/1756-0500-3-105 (PMC2862038; doi:10.1186/1756-0500-3-105)
Supplement: Additional file 2 — Michigan Salmonella case-control study, 2007: Enrollment of cases (12/15/06 - 10/15/2007). All cases of children 10 years or younger in Michigan who were confirmed by laboratory methods to be a case of non-typhoidal Salmonella infection and signed a consent form, were enrolled. [file 1756-0500-3-105-S2.DOC]

**Figure S1.** Michigan *Salmonella* case-control study, 2007: Enrollment of cases (12/15/06 - 10/15/2007)

All reported cases between 12/15/06 and 10/15/2007

**n=862**

Incomplete address/phone number

**n=29**

(29/228)=12.72%

Declined to participate

**n=10**

(10/169)=5.91%

Comorbid condition

**n=1**

(1/170)=0.59%

Answering machine/busy call /could

not be contacted at all

**n=36**

(36/159)=22.64%

Phone interview

**n=102**

(102/123)=82.94%

Cases in children

aged ≤10 years

**n=228**

Non-typhoidal cases in children aged ≤10 years

**n=199**

Typhoidal cases

**n=29**

(29/228)=12.72%

Invitation letter sent

**n=170**

Eligibility criteria met

**n=169**

Written or oral consent provided

**n=159**

Interviewed

**n=123**

(123/169)=72.35%

Mail-in-Questionnaire

**n=21**

(21/123)=17.06%

Cases in aged >11 years

**n=651**

(651/862)=75.52%
