# Supplementary material for: Case-control study of disease determinants for non-typhoidal Salmonella infections among Michigan children
Source: BMC Res Notes. 2010 Apr 16;3:105. doi: 10.1186/1756-0500-3-105 (PMC2862038; doi:10.1186/1756-0500-3-105)
Supplement: Additional file 3 — Michigan Salmonella case-control study, 2007: Enrollment of controls (12/15/06 - 10/15/2007). All non-case children who were matched on age and gender with cases and signed a consent form were enrolled. [file 1756-0500-3-105-S3.DOC]

Figure S2. Michigan Salmonella case-control study, 2007: Enrollment of controls

(12/15/06 - 10/15/2007)

Controls

**Method-2**

**Method-1**

Total phone numbers obtained

n=2,463

Potential controls obtained from case parents

n=37

Disconnected phone numbers

n=445

(445/2463)=18.07%

Valid phone numbers

**n=2,018**

Commercial phone numbers

n=53

(53/2463)=2.15%

Household phone

numbers

**n=1,965**

Could not be contacted again/refused

n=9

(9/37)=24.32%

Answering machine

n=1134

(1134/2463)=46.04%

Received phone call

**n=831**

Hung up/not interested

n=338

(338/2463)=13.72%

Screened for potential control children

# n=493

No children or children <10 years

n=371

(371/2463)=15.06%

Scheduled interview

# n=122

Could not be contacted again

n=11

(11/2463)=0.45%

Interviewed

# n=111

(111/2463)=4.51%

Interviewed

n=28

(28/37)=75.68%
